# The Royal Sea-Bathing Hospital, Margate

**Published:** 1902-03-29

**Authors:** 


### THE ROYAL SEA-BATHING HOSPITAL, MARGATE.

### SOME CRITICISMS.

Several questions were raised by the Governors of the above hospital on Monday afternoon, March 24th, when the annual meeting took place at 30 Charing Cross, the London office. Or, rather, two Governors raised the questions, two or three more were present, and the rest did not put in an appearance.

Mr. Biddulph, the Chairman, being abroad, the chair was taken by Mr. F. C. Norton, a member of the Court of Directors, who went over the main points of the report and accounts, and invited criticisms before moving their adoption.

### A Question oe Expense.

Mr. Barker Young said he would like to ask a question with regard to the circulation of the report. The Chairman had said that it was now in the hands of the Governors; did he mean by "now" that moment? Mr. Young had arrived twenty minutes before the time, with the intention of looking through the report, but the committee was sitting and he could not get a copy.

The Chairman apologised. He said there was a custom for a great many years of sending out a draft report to all the governors before the meeting; he thought it was done until about two years ago, when it was dropped on account of the heavy expense. Since then only a certain number of draft copies had been printed, and though a notice was sent to all the Governors, the draft report was sent only to a few. It would be sent out after the meeting, as soon as the committee had had time to consider some alterations in the rules. Mr. Barker Young also suggested that the notice convening the meeting should be signed by somebody as it was, a bare notice was received such as anyone might send.

The Chairman promised that this should be seen to.

Mr. Young further objected that, though it was stated that the copy of the proposed alterations might be seen at the office, he had been told, on inquiring for it, that there were only two copies, and he did not like to disturb the Court to ask for one of them.

The Chairman said he was very sorry. He moved the adoption of the report.

### Wider Recognition.

Mr. Sankey seconded. He said how very much surprised he was to find only half-a-dozen Governors present. He should have thought a big meeting might have been organised. He had known the hospital all his life, and there was an opinion amongst many people that it ought to be better known. He considered it a very great pity that the meeting was organised in a room not large enough to hold many people. The Chairman had spoken of having to shut up some of the beds, and of the want of financial support, and so forth. He could not help thinking there was an alternative to this state of affairs. The Governors' names filled 40 closely printed pages of the report. Without wishing, as a new governor, to throw any blame, he would like to suggest that some means be taken to bring the hospital before the public; it ought to have a wider recognition.

### The Usual Thing.

Mr. Baker Young said: "It is the usual thing. I happen to be a governor of one of the London hospitals, and I have been at a meeting when three or four only were present. I don't know that annual meetings do very much good, but I certainly think the public should get the information through the Press. I don't think you will get many to an annual meeting; as long as the Governors are satisfied they will not come. Depend upon it, if you don't do the business properly you will very soon get a full room. As long as things go on well, they are contented, and leave it in your hands. I don't agree," Mr. Young added, "about the change of rule with regard to the appointment of medical officers; how long have you tried it?"

### The Appointment of Medical Officers.

The Chairman: A very short time. We have had very great difficulty in getting young men to take up the work.

A discussion followed on the question of medical appointments, which have just been reduced at Margate to six months' junior followed by six months' senior residence. Mr. Young said other hospitals had tried it, and had found that it worked well to hold out the hope of preferment, but he thought the committee should leave themselves a loophole in case a man proved suitable and they wished to retain him. He had noticed in the columns of a medical journal that the Margate Hospital had advertised, week after week, for a very long time.

The Chairman said that young and ambitious men went to South Africa; there had been the greatest possible difficulty in getting a duly qualified man to take the work.

Mr. Young insisted on the necessity of leaving a loophole for the committee to continue the appointment if desirable.

### The Auditors.

The next question was that of the Auditors. The fee was stated to be 30 guineas; this, Mr. Young said, was too much; there were plenty of firms who would do it for 20 for a charity. The Chairman said the accounts were somewhat complicated on account of having the hospital in one place and the office in another. Finally, the re-election of the auditors was allowed to stand over, inquiries to be made in the meantime with a view to getting the work done for a smaller fee, the Governors agreeing to leave the matter in the hands of the Court.

A vote of thanks to the bankers being proposed, Mr. Young expressed his pleasure in seconding it, since no interest was charged on loans.

The Chairman said that an amicable arrangement had been made with the tramways company, and there was no longer any fear of the hospital's sea-wall breaking down.

### A Special Meeting.

The meeting, up to this moment a general one, then became special, to consider the alteration of rules.

Mr. Young objected to the proposal to substitute two for three as the quorum for a Court. It would be very damaging if it were known that the work was going to be done by two persons.

The Chairman said it had been found impossible, even with three, to get a quorum, but he agreed to drop the alteration.

The question of the six months' appointment came up again, and Mr. Young said he would like to ask Mr. Willett, who had just arrived, what he thought about it. Mr. Willett was of the opinion that you must hold out a sufficient inducement to a young man; if you retained the senior medical officer, the junior had no chance; he remained a junior. The junior doctors had to dispense, and they did not object to it for six months if there were a prospect of promotion at the end of that time. It was felt that it would work better, and would bring the Margate Hospital more into line with what was done in the London hospitals.

No other disputed alterations were brought forward, and the meeting broke up with votes of thanks, Mr. Young remarking on the patience exhibited by the Chairman under his somewhat critical remarks.

Besides those already mentioned, General Campbell and Dr. Mackenzie were present.

